# A comparative analysis of physical fitness of children and adolescents with HIV infection: A systematic review protocol

**DOI:** 10.1097/MD.0000000000018206

**Published:** 2019-12-10

**Authors:** Rafaela Catherine da Silva Cunha de Medeiros, Isis Kelly dos Santos, Carlos Jean Damasceno de Goes, Anna Luiza Vasconcelos de Oliveira, Jason Azevedo de Medeiros, Ricardo Ney Cobucci, Paulo Moreira Silva Dantas

**Affiliations:** a Postgraduate Program in Health Science, Federal University of Rio Grande do Norte; b Department of Nutrition, Federal University of Rio Grande do Norte; c Department of Physical Education, Federal University of Rio Grande do Norte; d Postgraduate Program in Biotechnology, Potiguar University, Natal, Rio Grande do Norte, Brazil.

**Keywords:** adolescents, children, HIV, physical fitness

## Abstract

**Background:**

The impaired physical capacity of children and adolescents with HIV can directly influence their physical performance, activities of daily living and social participation. The purpose of this systematic review protocol is to perform a systematic review and meta-analysis on physical fitness (cardiorespiratory capacity, agility, flexibility, strength, and muscular endurance) in children and adolescents with HIV, compared with healthy controls.

**Methods:**

We will be following the Preferred Reporting Items for Systematic Reviews and Meta-Analysis protocol (PRISMA-P) statement guidelines. There will be cross-sectional, longitudinal and case-controlled studies, and there will be no restrictions on language and year of publication in the search. The search strategy will be to use databases including: MEDLINE (via PubMed), EMBASE (via Ovid), Web of Science, Scopus, SportDiscus and CINAHL; The MeSH terms will be: physical fitness, fitness trackers, agility, flexibility, physical endurance, muscle strength, aerobic capacity, human immunodeficiency virus, HIV, children, and adolescents, to discuss and compare physical fitness (cardiorespiratory capacity, agility, flexibility, strength, and muscular endurance) in children and adolescents with HIV and healthy control. The reviewers will independently read the articles, extract the data information and analyze the risk of bias using the Cochrane criteria for observational studies. The Cohen's will be used to calculate the agreement between the revisions.

**Results:**

This study will provide a high-quality synthesis of observational studies on the analysis and comparison of physical fitness in children and adolescents with HIV compared with healthy controls.

**Conclusion:**

This systematic review will be very important for the creation of proposals aimed at providing high quality subsidies in the management of HIV during the development phase of children and adolescents.

**Ethics and dissemination:**

Ethics approval is not required because individual patient data and privacy were not involved in this study.

**PROSPERO registration number:**

CRD42019140955.

**PROSPERO registration date:**

23/09/2019.

## 1 Introduction

The Human Immune Virus (HIV) is a chronic disease that promotes various consequences such as metabolic and neurological abnormalities in body composition, amongst others. Together with the use of prolonged antiretroviral therapy (ART), these complications intensify and are a serious and continuous problem in young people infected with HIV, especially in the condition of vertical transmission.^[1,2]^

It is evidenced in the literature that the physical fitness of children and adolescents living with HIV is impaired, because lower values are found in agility, flexibility, strength, power, muscular endurance, cardiorespiratory aptitudes (lower VO2 peak) and body composition (lower mean height, body mass and BMI; body fat accumulation in the abdominal region).^[3–5]^

The physical capacity of impaired HIV-infected children and adolescents can directly influence physical performance and daily life activities, which in turn influences social participation, as these complications are associated with other psychosocial challenges that impact on their ability to live with the HIV disease.^[6]^ There are also reports of lower physical activity score.^[7]^ Especially in the context of sport and physical activity, this population with HIV needs to take care to minimize risk factors for the development of cardiovascular disease, and focus on health promotion, disease prevention, and treatment adherence strategies, which occur in the school environment or in the facilities. Basic health services have the potential to reduce stigma-related problems against discrimination, which consequently affects collective activities that directly interfere with the socialization of this population.^[8]^

Despite some evidence, studies with children and adolescents with HIV that evaluate all the parameters related to health and performance are rare. Some studies have been published, however, only 1 systematic review has been published comparing the physical fitness of adult people with HIV with healthy control subjects.^[2]^ Thus, there is a need to emphasize these factors in the pediatric population, as it is a population that deserves attention.

### 1.1 Review questions

The objective of this systematic review protocol is to conduct a systematic review and meta-analysis about the physical fitness (cardiorespiratory capacity, agility, flexibility, strength, and muscular endurance) in children and adolescents with HIV and compare with healthy controls. The question was structured or refined using P(E)COS: P- children and adolescents; E- HIV; C- uninfected; O- physical fitness (cardiorespiratory capacity, agility, flexibility, strength and muscular endurance); S- observational studies.

## 2 Methods

The Preferred Reporting Items for Systematic Reviews and Meta-Analysis Protocol (PRISMA-P) guidelines for systematic review and meta-analysis were followed.^[9]^ The study was registered in PROSPERO and assigned the registration number: CDR42019140955.

### 2.1 Selection criteria

Studies that meet the following criteria will be included in the systematic review:

#### 2.1.1 Inclusion criteria

1. Studies that are cross-sectional, longitudinal and case-controlled, with no restriction of languages and year of publication;
2. Studies that discuss and compare physical fitness (cardiorespiratory capacity, agility, flexibility, strength, and muscular endurance) among children and adolescents with HIV and healthy controls.
3. Appoint changes in physical fitness or general values between groups as defined in the studies, measured through validated tests;

#### 2.1.2 Exclusion criteria

1. Case reports, case studies, letters to the editor, fact sheets, conference abstracts, and review articles;
2. Studies investigating adult populations (>18 years).

### 2.2 Search strategy

The studies published will be performed in databases which include: MEDLINE (via PubMed), EMBASE (via Ovid), web of science, Scopus, SportDiscus and CINAHL. The reference lists will be screened. The search strategy will be to use the medical subjective headings (MeSH) and terms that have been included in Table 1. The literature screening will be performed by 2 reviewers.

**Table 1:**
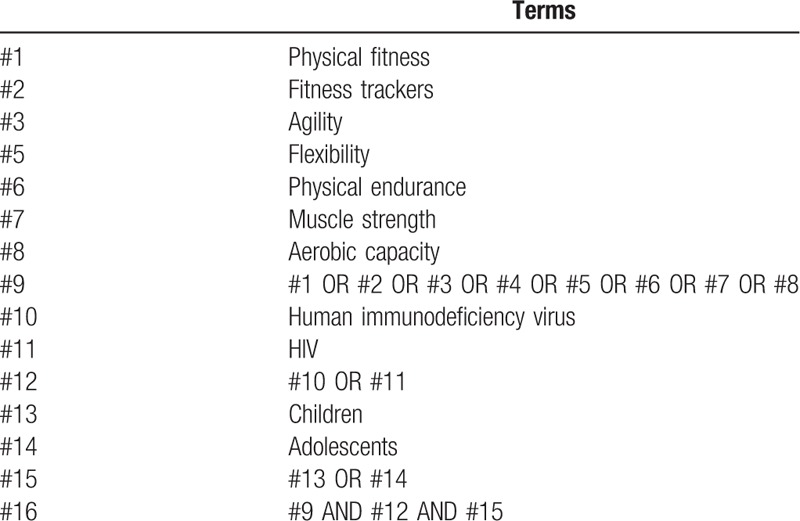
Search strategy – MeSH terms.

### 2.3 Study selection

**Figure 1:**
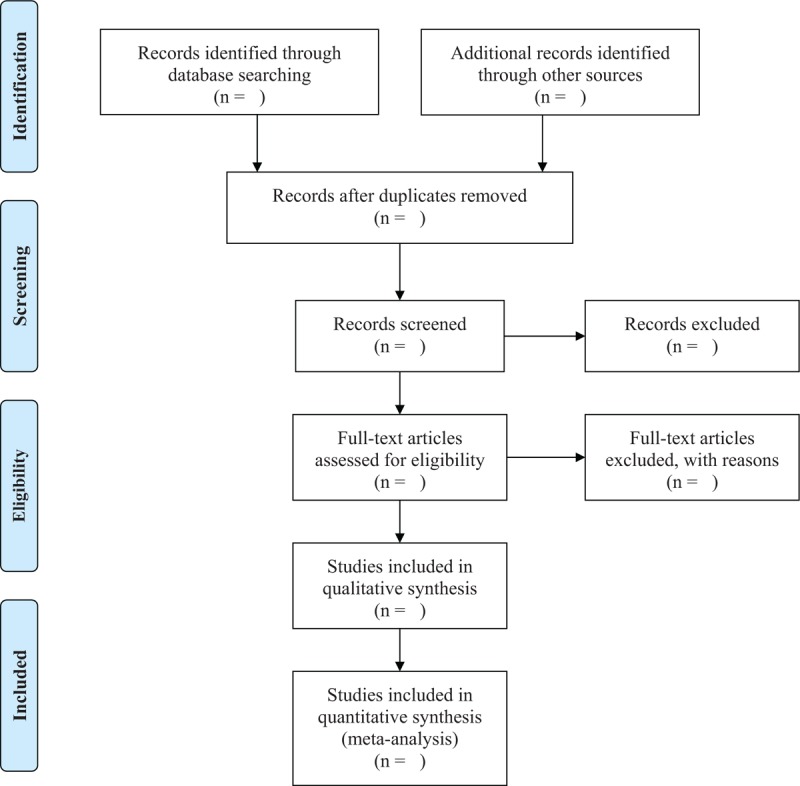
Flow diagram of study selection process. n = number.

The studies identified will be stored in a citation management software (EndNote - Thompson Reuters, CA). All studies identified through search strategies (titles and abstracts) will be independently screened by 2 authors based on the inclusion criteria described above (Level 1). Potentially eligible studies will be researched again by reading the full text (Level 2). In the case of disagreement between reviewers, a third review author will adjudicate the final decision. We will use the Rayyan QCRI (Rayyan QCRI software, Qatar Computing Research Institute (Data Analysis), Doha, Qatar)^[10]^ to remove duplicates, and for screen citations at title and abstract (Level 1) and full text (Level 2) and extract data. Corresponding authors of studies unavailable for full reading will be contacted via email. The entire study selection process will be specified in the PRISMA flow diagram found in Figure 1.^[11]^

### 2.4 Assessment of methodological quality

Selected studies will be assessed for methodological quality used a 3-domain scale (selection bias, measurement, outcome, and reporting bias) with 14 items. This tool is based on other studies with the same population that used the Cochrane criteria for observational studies and the NIH tool for observational and cohort studies and cross-sectional studies.^[12,13]^

The scale determines how each study addressed questions related to the research question; study population; appropriate sample size; Groups recruited from the same population and with uniform eligibility criteria; Justification of sample size; Presentation of results; Deadline to analyze effect; Differences between exposure levels of interest; Exposure and assessment measurements; repeated exposure assessment; Outcome measures of Blindness for outcome evaluators; Follow-up rate and Statistical analysis.

For the final analysis of the quality scores of the 14 items, the values are divided into 14 points, where values below 7 points indicate high risk, between 7 and 10 moderate risk and above 10 points shows a low risk of bias. Two independent reviewers will assess the studies and rate each criterion yes, no, or other (not clear). If there is any conflict in the assessment a third reviewer will complete the reviews. We will use the Cohen k calculation to assess the agreement among reviews.

### 2.5 Data collection and extracted

Data will be carefully extracted using a standard form covering topics such as: year of publication, (b) study design, (c) location, (d) participant characteristics (age, gender, sample size), (e) use of medications, (f) main outcomes and instruments used (components of physical fitness), (g) control variables (sociodemographic, clinical, and physical activity level variables) and (h) additional outcomes and adverse events. For research data that are not clear or present any difficulty at the time of extraction, the corresponding author will be contacted for possible clarification. Two authors will independently extract the data.

### 2.6 Data synthesis

We will determine the possibility of conducting a meta-analysis based on measurement methods and heterogeneity. Dichotomous data from each of the eligible studies will be combined for meta-analysis. Results will be expressed as Odds Ratios (OR) with 95% Confidence Intervals (CI). Fixed-effects or random-effects models will be chosen depending on whether there will be an absence or presence of heterogeneity between studies. All tests will be performed using Review Manager (RevMan version 5.3.0) software and a two-sided *P* value of <.05 will be considered statistically significant. Determination will be included in the review, if it makes sense to combine data, using the I^2^ statistic (<25%, without heterogeneity, 25%–50%, moderate heterogeneity, and >50% strong heterogeneity).^[14]^ In addition, when possible we will use Egger's funnel chart to evaluate possible publication biases.^[15]^ The data will be entered into the Review Manager software (RevMan 5.3). Meta-regression will be conducted to investigate the potential heterogeneity.

### 2.7 Main outcome(s) and Additional outcome(s)

The pre-specified main (most important) outcomes of the review are: Changes in physical fitness defined in the studies, measured through tests validated as: the body composition (Dual energy X-ray absorptiometry - DXA and Anthropometry); Cardiorespiratory (modified Balke and Bruce protocol); Agility (Shuttle Ru test); Flexibility (the modified sit and reach test); and Strength and muscular endurance (1-Repetition Maximum – 1RM, with dynamometry and tests of vertical thrust). The pre-specified additional outcomes of the review are: Changes in physical activity level (PAL) measured through tests validated as: Pedometer, accelerometer and questionnaires (Physical Activity Questionnaire - PAQ-C and International Questionnaire of Physical Activity - IPAQ).

## 3 Discussion

Previous systematic reviews have shown data on the impairments in physical fitness caused by the HIV virus and the use of antiretroviral therapy in adults living with HIV compared with healthy peers.^[13]^ In this sense this protocol is the first systematic review to evaluate the physical fitness (cardiorespiratory capacity, agility, flexibility, strength, and muscular endurance) in children and adolescents with HIV compared with healthy controls. There is a predominance of impairments in physical capacity in the pediatric population with HIV, and it is evident that there is the need to conduct a systematic review in order to provide in-depth details on the subject, as well as to observe the limitations and alterations caused in the growth and motor development of the pediatric population.

Investigating the physical capacities of healthy children and adolescents will help us to understand the virus's ability to increase deficits in the pediatric population with HIV, as well as to assist in future interventions that will complement the treatment in childhood. This systematic review will present important evidence on the subject, as well as enable the construction of current evidence that will generate significant results in future forms of treatment, care and health policy. It will assist health professionals (physicians, physical education professionals, nurses, etc) from the pedagogical areas in the handling of future interventions and decision making in order to help in the treatment of this population.

## Author contributions

**Conceptualization:** Rafaela Catherine da Silva Cunha de Medeiros; Paulo Moreira Silva Dantas.

**Data curation:** Rafaela Catherine da Silva Cunha de Medeiros; Isis Kelly dos Santos.

**Formal analysis:** Rafaela Catherine da Silva Cunha de Medeiros; Isis Kelly dos Santos.

**Investigation:** Rafaela Catherine da Silva Cunha de Medeiros; Isis Kelly dos Santos; Anna Luiza Vasconcelos de Oliveira; Carlos Jean Damasceno de Goes.

**Methodology:** Rafaela Catherine da Silva Cunha de Medeiros; Isis Kelly dos Santos; Anna Luiza Vasconcelos de Oliveira; Carlos Jean Damasceno de Goes; Ricardo Ney Cobucci.

**Writing – original draft:** Rafaela Catherine da Silva Cunha de Medeiros; Isis Kelly dos Santos; Jason Azevedo de Medeiros.

**Writing – review and editing:** Rafaela Catherine da Silva Cunha de Medeiros; Isis Kelly dos Santos; Ricardo Ney Cobucci.

**Supervision:** Paulo Moreira Silva Dantas; Ricardo Ney Cobucci.

**Methodology:** Ricardo Ney Cobucci.

**Supervision:** Ricardo Ney Cobucci.

**Writing – original draft:** Ricardo Ney Cobucci.

**Writing – review & editing:** Ricardo Ney Cobucci.

Rafaela Catherine da Silva Cunha de Medeiros orcid: 0000-0003-2150-5190.
